# Lysophospholipid Supplementation in Broiler Breeders’ Diet Benefits Offspring’s Productive Performance, Blood Parameters, and Hepatic β-Oxidation Genes

**DOI:** 10.3390/ani14213066

**Published:** 2024-10-24

**Authors:** Mohammad Sedghi, Fatemeh Javanmard, Anvar Amoozmehr, Saeid Zamany, Ishmael Mohammadi, Woo Kim, Venkata Sesha Reddy Choppa

**Affiliations:** 1 Department of Animal Sciences, College of Agriculture, Isfahan University of Technology, Isfahan 84156-83111, Iran; fjavanmard.12174@gmail.com (F.J.); saeidzamany@ag.iut.ac.ir (S.Z.); esmail.mohammadi@ag.iut.ac.ir (I.M.); 2 Department of Animal and Poultry Nutrition, Faculty of Animal Science, Gorgan University of Agricultural Sciences and Natural Resources, Shahid Beheshti Ave, Gorgan 49138-15739, Iran; anwaramoozmehr@gmail.com; 3 Department of Poultry Science, University of Georgia, Athens, GA 30602, USA; wkkim@uga.edu (W.K.); vc68270@uga.edu (V.S.R.C.)

**Keywords:** lysophospholipid, metabolizable energy, hepatic β-oxidation, *PGC-1α*, *LPCAT3*, gene expression

## Abstract

**Simple Summary:**

The growth and vitality of bird embryos during incubation are solely dependent on the nutrients deposited in the eggs. Lysophospholipid, a type of emulsifier, has been included in poultry diets to promote lipid digestion and absorption. This research aimed to explore the potential benefits of adding lysophospholipid to the diet of broiler-type breeders on their offspring. Four test diets were formulated with two energy levels and two levels of lysophospholipid supplementation. The experimental diets were fed to a total of 264 49-week-old breeder hens for periods of 8 and 12 weeks, and the hatched chicks were raised and evaluated at the age of 7 days. In summary, adding lysophospholipid (LPL) to the breeders’ diet led to improved offspring body weight, a better food conversion ratio, and lower blood fat levels. Additionally, it supported liver health and increased the expression of genes associated with lipid processing in both the gut and liver.

**Abstract:**

The present study aimed to investigate whether supplementation of modified lysophospholipids (LPLs) in the diet of broiler breeders can benefit their offspring. A total of 264 49-week-old breeders (Ross 308) were allocated and fed based on a 2 × 2 factorial arrangement with two levels of dietary energy (normal energy = 2800 kcal/kg and low energy = 2760 kcal/kg) and two LPL levels (0 and 0.5 g/kg) for periods of 8 and 12 weeks. The offspring were assessed for growth performance, serum parameters, hepatic antioxidative capability, and expression of genes involved in liver β-oxidation at 7 days old. The LPL inclusion improved (*p* < 0.01) average body weight (ABW), average daily gain (ADG), and feed conversion ratio (FCR). The offspring of 61-week-old breeders fed with LPL exhibited reduced serum triglyceride levels (*p* < 0.01) but an increase in hepatic glutathione peroxidase (*p* < 0.05). The LPL increased (*p* < 0.001) the mRNA expression of the *PGC-1α* gene in the liver. Supplementing LPL in low-energy diets resulted in higher *FABP1* gene expression (*p* < 0.05) in the intestine. In conclusion, LPL supplementation in the breeders’ diet improved offspring performance by enhancing fatty acid absorption, hepatic indices, and the expression of genes involved in liver β-oxidation.

## 1. Introduction

Maternal nutrition has a significant impact on the phenotype of offspring [1]. In birds, embryonic development during incubation depends entirely on the nutrients deposited in the eggs [2]. Nutritional changes in the chick embryo may cause epigenetic modifications, altering gene expression, structure, and function of organs and tissues in offspring [2,3]. It has been reported that the supplemental fatty acids (FA) in the breeders’ diet increased the incorporation of dietary FAs into the egg yolk and, therefore, affected the progeny’s development during incubation [4]. In addition, the FAs in the egg yolk were transferred into the liver of the offspring through the yolk residue by the time of hatching [4,5]. This suggests that the FA composition of the embryo’s tissue and the chicks that hatch rely on the fatty acid profile of the egg yolk [6]. This led us to the concept of maternal supplementation of lysophospholipid (LPL) in the broiler breeders’ diet and its subsequent impact on the offspring.

Exogenous emulsifiers such as LPLs have been included in the diet to promote lipid digestion and absorption in poultry [7,8]. LPLs increase the active surface area of lipids by integrating fatty acids into micelles, allowing the lipase enzyme to efficiently hydrolyze triglyceride molecules into fatty acids and monoglycerides. This process leads to increased dietary lipid digestibility in the duodenum [9,10]. In addition, the integration of LPLs into the phospholipid bilayer of enterocytes increases the permeability and formation of ion channels, resulting in an increased influx of micro and macromolecules through these cells [11,12,13,14]. Altogether, the contribution of LPLs to lipid emulsification coupled with cell membrane modification may lead to improved nutrient absorption [15,16].

By modulating genes involved in β-oxidation, LPL regulates the mechanism of fatty acid utilization and influences tissue inflammation. Specifically, LPL reduces hepatic fatty acid utilization through the downregulation of *PPARγ* (Peroxisome proliferator-activated receptor gamma) transcription [17] and downstream factors such as the *PGC-1α* (Peroxisome proliferator-activated receptor gamma coactivator 1 alpha) gene, which is involved in the β-oxidation pathway in hepatic cells [18]. The lipoprotein lipase enzyme facilitates lipolysis and the delivery of fatty acids to apolipoproteins for oxidation in various cell types [19]. Once fatty acids and phospholipids within apolipoproteins enter the cytoplasm of cells, they undergo acylation through LPCAT3 (Lysophosphatidylcholine Acyltransferase 3) [20]. Additionally, carnitine palmitoyl transferase 1 (CPT1), produced by the expression of the *CPT1A* gene, plays an important role in hepatic triglyceride metabolism. It catalyzes the transfer of the acyl group of long-chain fatty acid-CoA conjugates onto carnitine [21].

It has been reported that the supplementation of LPLs in the diet increases the apparent metabolizable energy and nitrogen retention in broilers [22,23,24,25]. Additionally, subsequent studies [9,25,26,27,28,29] have confirmed that dietary supplementation of LPL positively affected the average daily gain (ADG) and feed conversion ratio (FCR) of broiler chickens fed low-energy diets. Other researchers have also observed the benefits of LPL supplementation for maintaining performance in broilers fed with low energy [15] and low-nitrogen diets [16,25,30]. Furthermore, LPLs such as Lysophosphatidylcholine (LPC) could potentially result in the up-regulation of the pro-inflammatory factors as well as *TGF-β* and *NF-κB*, leading to the migration of monocytes and macrophages to the gastrointestinal wall [31]. 

Despite their beneficial effects, there is limited information available on the effect of LPL inclusion in the broiler breeders’ diet and its subsequent impact on the progeny. On the other hand, the mechanisms involved in lipid metabolism are influenced by many factors, such as breeders’ age [32,33], the energy level of the diet [34,35], and the duration of LPL inclusion. Therefore, the potential effect of dietary energy and the duration of LPL inclusion was taken into account. The current study aims to investigate whether LPL supplementation in the breeders’ diet can benefit newly hatched chick performance, serum parameters, the antioxidative capacity of the liver, and the downstream gene expression changes involved in both hepatic β-oxidation pathway and fatty acid absorption.

## 2. Materials and Methods

### 2.1. Bird Husbandry and Dietary Treatments

The experimental procedure was approved by the Guidelines for the Care and Use of Laboratory Animals in Iran [36]. A total of two hundred and sixty-four 49-week-old Ross 308 broiler breeders (240 hens, average body weight 3810 g ± 165 SD, and 24 roosters, average body weight 4690 g ± 181 SD) with similar physiological status were chosen and randomly subjected to experimental treatments for 12 weeks. Birds were placed in a floored house and kept according to the Ross 308 breeder management guide [37]. 

Breeders were randomly allocated to a 2 × 2 factorial arrangement design with two levels of dietary energy (normal energy = 2800 kcal/kg and low energy = 2760 kcal/kg) and two LPL supplement levels (0 and 0.5 g/kg), and 6 replicates (36 pens in total, each containing 10 hens and 1 rooster).

The dietary treatments were as follows: 1—Normal energy diet (NE, metabolizable energy = 2800 kcal/kg) as the control group; 2—Normal energy diet supplemented with LPL 0.5 g/kg (NE + LPL); 3—Low energy diet (LE, metabolizable energy = 2760 kcal/kg); and 4—Low energy diet supplemented with LPL 0.5 g/kg (LE + LPL).

The LPL supplement (Lipidol^®^, Pathway Intermediates, Seoul, Republic of Korea) was obtained from soybean lecithin using an exclusive proprietary technology). According to the manufacturer, each kilogram of this lysophospholipid product has a matrix value of 80,000 kcal/kg of ME. The recommended dosage is 0.5 g per kg of feed, which contributes a total of 40 kcal per kg of feed. 

On the last 7 consecutive days of weeks 8 and 12 of the experiment, the eggs laid by the breeders were collected twice a day, grouped by pen, and stored in a temperature-controlled room at 16 °C. From each experimental group, a total of 120 high-quality eggs were selected and sent to the hatchery. These eggs were hatched under the ambient conditions of 60 to 65% relative humidity and 37.8 °C temperature, with intermittent rotation. After hatching, the chicks were transferred to the experimental farm in two separate batches (weeks 8 and 12). The healthy chicks, with an average initial body weight of 44 ± 0.5 g, were then transferred to the Isfahan University of Technology research center and placed in a caged house.

In the first hatch (week 8 of the experiment), 86, 92, 93, and 90 chicks (361 chicks in total) were hatched from NE, NE + LPL, LE, and LE + LPL groups, respectively. The healthy chicks were randomly selected and assigned to 36 cages, allocated into 4 groups according to their maternal dietary treatment with 9 replicates (cages) of 10 birds each, and reared for 7 days to assess their performance. In the second hatch (week 12 of the experiment), 92, 93, 104, and 98 chicks (387 chicks in total) were hatched from NE, NE + LPL, LE, and LE + LPL groups, respectively. Similarly, these chicks were randomly selected and assigned to 28 cages, allocated into 4 groups according to their maternal dietary treatment with 7 replicates (cages) of 10 birds each, and reared for 7 days to assess their performance. When assigning chicks, it was ensured that all replicates within each treatment had similar body weights. The remaining offspring of the second hatch were kept separately for assessment of serum parameters, hepatic antioxidative capability, and expression of genes involved in liver β-oxidation at 1 day of age. Chicks from both hatches were raised under the same condition for a week, following the rearing guideline of the Ross 308 broiler management guide [38]. A lighting program of 23 h light plus 1h darkness was applied throughout the experimental period. The house temperature was initially set at 33 °C upon the arrival of the chicks and gradually decreased by 0.5 °C each day until it reached 30 °C on day 7 of the experiment. Chicks were fed a control diet (Table 1) formulated in accordance with the Ross 308 nutrient specifications [39]. The diet was provided using trough feeders, and the chicks had unlimited access to water. At the end of the experimental period, the individual weight of each chick and the total feed intake of each cage were recorded to determine the average daily gain (ADG) and average daily feed intake (ADFI). The feed conversion ratio (FCR) was calculated using the formula: FCR = ADFI (g)/ADG (g). These calculations were performed separately for each cage.

### 2.2. Sample Collection

On day one, a total of 28 chicks (7 chicks per treatment) hatched from 61-week-old breeders were randomly selected and humanely euthanized by CO_2_ inhalation for sample collection. Blood samples were collected via cardiac puncture by inserting a heparinized needle through the heart ventricle [40,41,42]. The needle was then removed from the syringe, and the blood was slowly aspirated into micro tubes, followed by centrifugation at 2000× *g* at 15 °C for 10 min to separate plasma from blood cells [43]. The plasma samples were stored at −20 °C prior to blood profile measurements. To measure the tissue gene expression, tissue samples were taken from the left lobe of the liver, pancreas, and jejunum (a longitudinal segment 5 cm anterior to the Meckel’s diverticulum). All samples were carefully washed with distilled water, immediately frozen in liquid nitrogen, and stored at −80 °C for subsequent analysis. In addition, the entire right lobe of the liver was collected and stored at −20 °C to measure the hepatic antioxidative capability.

### 2.3. Plasma Parameters and Liver Tissue Analysis

The plasma samples were analyzed for triglyceride (TG), cholesterol, albumin, aspartate aminotransferase (AST), alanine aminotransferase (ALT), and alkaline phosphatase (ALP). Furthermore, the liver tissue samples were minced and homogenized in an ice-cold 0.9% phosphate-buffered saline solution using a homogenizer (Ultra-Turrax^®^, IKA Works, Inc., Wilmington, NC, USA). Afterward, the mixture was centrifuged at 4000 RPM for 10 min at 4 °C, and the supernatant was used for measurement of superoxide dismutase (SOD), glutathione peroxidase (GPx), total protein (TP), total antioxidative capacity (TAC), and malondialdehyde (MDA) content. Both plasma and liver tissue samples were analyzed using an automatic analyzer (Alycon 300i, Dual voltage instrument; Abbott Laboratories Ltd., Chicago, IL, USA) with commercial assay kits (Pars Azmun^®^ medical equipment manufacturing, Tehran, Iran).

### 2.4. Total RNA Extraction

The liver, jejunum, and pancreas tissues were placed separately into a stainless-steel grinding plate with appropriate steel beads, and 1 mL of TRIzol solution was added to each sample. The mixture was ground for 1–2 min. Next, the solution was transferred to a 1.5 mL microtube, and 200 µL chloroform was added to the suspension, followed by vortexing for 1 min. The solutions were then left at room temperature for 20 min and centrifuged at 13,000× g RPM for 15 min at 4 °C. The upper clear phase was transferred to another set of microtubes, followed by the addition of 500 µL isopropanol, and centrifuged at 11,500 RPM for 15 min at 4 °C. The supernatant was removed, and 1 mL of ethanol was added and vortexed until the RNA pellet was separated from the bottom of the microtube and centrifuged at 11,500 RPM for 10 min at 4 °C. Subsequently, the supernatant was removed, and the pellet was left to dry at room temperature. After ensuring that the ethanol has evaporated, diethylpyrocarbonate (DEPC)-treated water was added based on the amount of RNA pellet. RNase-free DNase I (Sinaclon, Tehran, Iran) was used to remove DNA contamination. The total quantity of RNA and purity ratios (260/280 ratios) were calculated using a NanoDrop-2000 (Thermo, Waltham, MA, USA). Finally, the extracted RNA was dissolved and stored at −80 °C for subsequent analysis.

### 2.5. Primer Design

All primers were designed using Primer 3 Plus online software (https://www.bioinformatics.nl/cgi-bin/primer3plus/primer3plus.cgi, accessed on 21 March 2021) and checked with the NCBI Primer Blast (https://www.ncbi.nlm.nih.gov/tools/primer-blast/, accessed on 21 March 2021) to confirm the correct targeting of the desired genes (Table 2). The primers were commercially purchased from TAG Co. Copenhagen, Denmark. 

### 2.6. Complementary DNA (cDNA) Synthesis

The cDNAs were synthesized using a random hexamer mix following the manufacturer’s instructions (cDNA Synthesis RT reagent Kit Sinaclon, Tehran, Iran). The resulting cDNA was stored at −20 °C for future experiments.

### 2.7. Quantitative Reverse Transcription Polymerase Chain Reaction (qRT-PCR)

The procedures and reagents for the qRT-PCR experiment were conducted as described in our previous publications [44,45,46]. Briefly, the cycle threshold (Ct) values of the triplicate PCRs were averaged, and the relative quantification of the transcript levels was performed using the comparative 2^−ΔΔCT^ method. The fold change in the target gene, relative to *GAPDH,* was determined according to the following formula: fold change = 2^−ΔΔCT^, where ΔΔCT = (Ct target gene − Ct *GAPDH*), ΔCT = CT (a target gene) − CT (a reference gene), ΔΔCT = ΔCT (a target sample) − ΔCT (a reference sample); every sample was further fortified without inverse transcription to ensure that no DNA impurity would be in the sample [47].

### 2.8. Statistical Analysis

Experimental data was analyzed using SAS software version 9.04 (SAS Institute, Cary, NC, USA) as a 2 × 2 factorial arrangement. Each cage served as the experimental unit. The statistical model used was: Yijk = µ + Mi + Lj + MLij + eijk, where Yijk represents the amount of each observation, µ is the experimental mean, Mi is the effect of metabolizable energy, Lj the effect of LPL supplementation, MLij is the interaction between metabolizable energy level and LPL supplementation level, and eijk is the error term. In simpler terms, the model comprised the main effects of the metabolizable energy level and LPL supplementation level, as well as the interaction between the metabolizable energy level and LPL supplementation level. The normality of the data was checked before conducting the ANOVA analysis. The significance was assessed using the two-way ANOVA analysis, and the Tukey test was used to differentiate between means. A *p*-value less than 0.05 was considered statistically significant. The data was presented as mean ± SEM for each group.

## 3. Results and Discussion

### 3.1. Broiler Performance

Previous research has shown that the level of diet metabolizable energy greatly affects the bird’s growth performance [35]. This energy is utilized by the animal for maintenance and production parameters [48]. Table 3 presents the performance of offspring during the 0–7 day period after hatching from breeders fed dietary treatments for 8 and 12 weeks (57 and 61-week-old). The interaction analysis revealed no significant difference across the dietary treatments. Moreover, the main effects showed that feeding the 57-week-old breeders with the normal energy diets resulted in offspring with superior (*p* < 0.05) ABW at the end of day 7, as compared to those fed the low energy diets. This suggests that offspring from the low-energy group primarily used energy for maintenance, limiting the energy towards production [48,49]. Previous studies have demonstrated that reducing the energy of a broiler diet significantly impaired birds’ ADG and FCR [25,30,50]. However, there is limited literature available to understand the impact of reducing the dietary metabolizable energy of breeders on the subsequent long-term performance of their offspring to confirm our results. Majdolhosseini et al. (2019) found that broilers fed a diet containing LPL 0.1% and 100 kcal/kg less dietary energy exhibited equivalent FCR and increased apparent digestibility of dry matter, nitrogen, ether extract, and gross energy at 24 d of age compared to the control group [50].

In our study, the ADG of chickens hatched from 57-week-old breeders fed with the low-energy diet tended to be lower (*p* = 0.07). Additionally, the inclusion of LPL resulted in improved (*p* < 0.05) ABW, ADG, and FCR in chickens hatched from both hens that fed with LPL for 8 and 12 weeks (57 and 61-week-old, respectively). However, feeding breeders for 12 weeks with 0.5 g/kg of LPL resulted in a greater ADFI of chickens (*p* = 0.06). These findings could be attributed to the role of the LPL supplement, which acted as an exogenous emulsifier, facilitating the digestion and absorption of lipids [51], and subsequently improved the offspring’s performance via increased fatty acid mobilization to the egg. The benefits of improved ADG and FCR in this study were consistent with the findings of Boontiam et al. (2019), who observed that the inclusion of 0.1% LPL in broilers’ diet could enhance growth performance in young birds from 1 to 21 d of age through the improvements in feed efficiency [16]. Similarly, Zhao and Kim (2017) reported improved growth performance and reduced FCR due to supplementation of LPL in broilers diet from 1 to 28 d of age compared to those fed the basal diet [25]. These findings highlight the vital role of modified LPL in lipid digestion, as it improves nutrient absorption by increasing micelle formation [52].

### 3.2. Blood Profile

Table 4 represents the serum biochemical parameters of day-old offspring of the second hatch, which fed dietary treatments for 12 weeks (61-week-old). The main effect of metabolizable energy showed that the low-energy diets resulted in decreased (*p* < 0.05) serum cholesterol compared to the normal energy diets. This is consistent with the findings of Boontiam et al. (2019), who reported reduced serum TG concentration in broilers fed 0.1% LPL supplement [16]. Similar results were detected by Hosseini et al. (2018), who stated that TG and LDL concentrations were decreased by the supplementation of 0.1% LPL in broilers fed with a low-energy diet on d 24 [53]. The faster absorption and metabolism rate of ingested fat may explain the lower serum TG levels in birds fed LPL [9,52]. This indicates that chylomicrons were either secreted into the serum at a slower rate or cleared from the blood at a faster rate [54], reflecting an improved lipid metabolism in the liver [55]. However, this effect needs further investigation since we did not measure the concentration of hepatic lipoproteins and lipid content in the blood and liver. Other possible mechanisms underlying the reduction in blood TG will be discussed using the expression of liver candidate genes. Interestingly, in the current study, the LPL supplement did not alter the serum cholesterol, which is in contrast to previous reports on broiler chickens [25,50,56]. These studies agreed that LPL supplementation in the diet significantly reduced cholesterol levels in serum.

In the present study, the offspring of breeders fed the low-energy diets had elevated (*p* < 0.05) serum AST compared to the normal energy diets. Feeding breeders with the LPL-containing diets resulted in chicks with a lower serum TG (*p* < 0.01) and AST (*p* < 0.01) compared to diets with no LPL supplement. Contrary to our findings, Boontiam et al. (2019) found that reduced metabolizable energy diets, either with or without LPL supplementation, did not significantly alter the AST enzyme activity in broilers. These authors concluded that an energy reduction of not more than 150 kcal/kg is safe for broilers [16]. Aspartate aminotransferase (AST) is a marker of mitochondrial activity in the Kupffer cells, and it is measured in serum to assess body metabolism rate and liver health status [57]. Higher serum AST activity indicates an increased rate of free amino acid utilization by catabolizing the amino acid carbon skeleton [58]. 

The analysis of output data showed no interactions between dietary treatments, except for the ALP, where the greatest serum ALP was related to the offspring of breeders who were fed the NE + LPL diet. Alkaline phosphatase (ALP) is a serological marker of bone metabolism, which provides a real-time assessment of bone formation, mineralization, and turnover. Although the exact function of ALP is unknown, it is mainly associated with increased osteoblastic activity in breeders and commercial chickens [59,60]. The increase in the ALP level of the NE + LPL group might be due to an increase in corticosteroids, epinephrine, and nor-epinephrine secretion [41]. Contrary to our findings, Lai et al. (2018) confirmed no significant difference in serum ALP activity of 42-day-old male broiler chickens fed with a high dose of bile salts (400 mg/kg) compared to the control group [61]. However, the mechanism responsible for these enzyme activity alterations is difficult to explain and needs further examination. It is crucial to acknowledge that in the present study, the total ALP was measured, which includes contributions from other sources, such as the liver. Therefore, future investigations should measure the bone-specific ALP to achieve a more comprehensive understanding.

### 3.3. Hepatic Antioxidative Capability 

Table 5 demonstrates the hepatic antioxidative capability of day-old offspring of the second hatched, which hatched from breeders fed dietary treatments for 12 weeks (61-week-old). SOD and GPx activity, as well as MDA and TAC levels, are used as markers of oxidant-antioxidant state in animals [62]. Superoxide dismutase (SOD) is a copper and zinc-containing enzyme that helps prevent the buildup of superoxide (O_2_^−^). The accumulation of superoxide can act as an oxidant by itself, and when combined with H_2_O_2_, it forms the OH radical, or it is combined with NO, which forms peroxynitrite [62]. Decreased or inhibited SOD activity may result in cellular membrane damage due to peroxidative processes initiated by the accumulation of free O_2_^−^ [63,64]. There were significant interactions between dietary energy and LPL supplementation on hepatic SOD (*p* < 0.01) and MDA (*p* < 0.05) levels in chickens. Interestingly, the addition of LPL to normal energy diets increased SOD and MDA levels, while its addition to low energy diets decreased SOD and MDA levels. This finding could be referred to as a higher demand for SOD enzyme for lipid metabolism, resulting from the higher absorption rate of fatty acids in the liver caused by LPL. In contrast, Siyal et al. (2017) observed a significant increase in hepatic SOD activity of 42 d old broilers fed with 0.1% soybean lecithin, as compared to the control group [65]. In this study, dietary LPL inclusion at 0.5 g/kg in breeders’ diet led to offspring with reduced hepatic SOD (*p* < 0.01) and increased hepatic GPx, indicating the improved antioxidant status of chickens [66]. Confirming the present findings, El-katcha et al. (2021) reported that feeding 60-d-old ducks with 0.05% lysolecithin resulted in increased GPx activity [67]. GPx predominantly catalyzes the conversion of H_2_O_2_ to H_2_O. Furthermore, GPx [68] catalyzes the reduction of fatty acid hydroperoxides, which are the primary oxygenated products of polyunsaturated fatty acids, as well as 1-monoacylglycerol hydroperoxides. 

MDA and T-AOC are widely used as indicators of oxidative stress in meat [69,70]. Malondialdehyde is a soluble degraded product of lipid peroxidation and an indicator of lipid oxidation intensity in tissues such as the liver, heart, kidney, spleen, lungs, egg, and erythrocytes. Elevated MDA levels in meat are associated with characteristics such as a rotten smell, loss of taste and color, and reduced nutritional value [71]. Our results suggest an increase (*p* < 0.01) in the offspring’s hepatic MDA level of breeders fed NE + LPL, which could be explained by the fact that the high-energy diet promoted performance in chickens, thus causing higher metabolic stress with an expected increase of MDA [72,73]. In Siyal et al. (2017) study, the hepatic MDA of birds fed with 0.1% soybean lecithin significantly reduced in comparison with the control group, which is in contrast with the findings of the current study [65]. Moreover, Wu et al. (2022) fed day-old goslings with 100 mg/kg soybean lecithin for 32 days and concluded that soybean lecithin supplementation led to a significant decrease in the serum MDA of birds [74].

### 3.4. Expression of Candidate Genes in the Liver 

To test whether the metabolizable energy levels and consumption of LPL by the breeders could influence the gene expression of offspring, we analyzed the expression of candidate genes in the liver, jejunum, and pancreas tissues.

We investigated the transcription of the *PPARγ* gene in the liver hepatocyte cells of the offspring broilers. There was no significant difference among treatments in mRNA expression for *PPARγ*. The *PPARγ* gene is responsible for triacylglycerol storage in the adipose tissue [74]. *PPARγ* gene expression was not affected by the treatments in the current study since the newly hatched chicks lack adipose tissue. 

**Figure 1:**
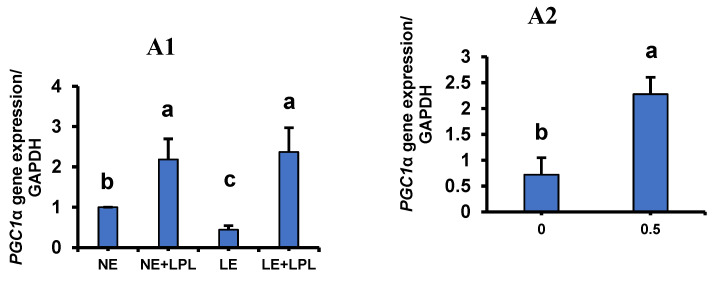

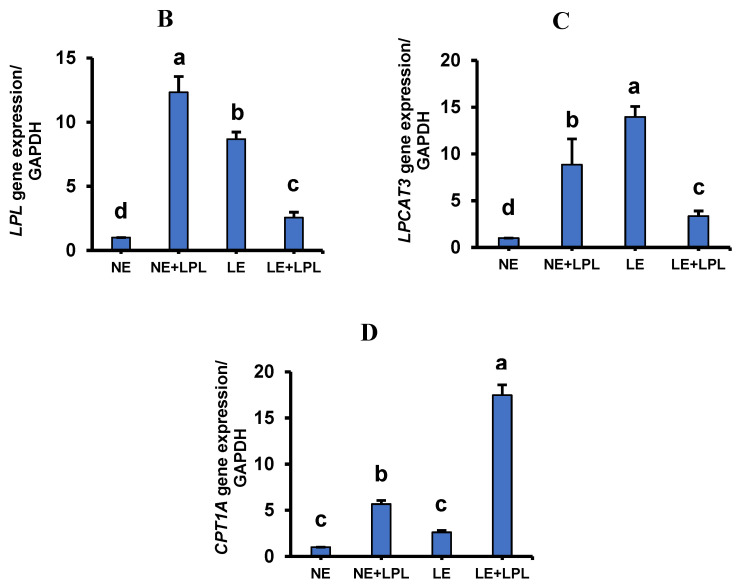
The interaction and the main effect of energy and LPL supplement on the transcription of the related genes with β-oxidation in the liver tissue. (**A1**) Peroxisome proliferator-activated receptor gamma coactivator 1-alpha (*PGC-1α*). (**A2**) The main effect of LPL supplement on the *PGC-1α* transcription. (**B**) Lipoprotein lipase *(LPL).* (**C**) Lysophosphatidylcholine acyltransferase 3 (*LPCAT3*). (**D**) Carnitine palmitoyltransferase 1A (*CPT1A*). Whiskers represent SEM. ^abcd^ Values within a column followed by different superscripts are significantly different. *p* < 0.05; Tukey’s pairwise comparison. NE: normal energy, LE: low energy, LPL: Lysophospholipid supplementation.

Reciprocal effects show that the LE diet led to a decrease in *PGC-1α* gene expression, but the LPL supplementation in both NE and LE diets increased the *PGC-1α* gene transcription. (*p* < 0.001). The NE + LPL and the LE + LPL groups showed an increase in *PGC-1α* expression, as compared to the NE and LE groups (*p* < 0.05). While the LE group showed a decrease (*p* < 0.05) in the *PGC-1α* expression when compared to the other groups (Figure 1(A1)). Several studies have shown that the *PGC-1α* gene is downstream of the *PPARγ* gene and, therefore, is affected by the expression of the *PPARγ* gene. The *PGC-1α* regulates key mitochondrial genes essential for adaptive thermogenesis and plays a crucial role in metabolic adjustments in response to dietary changes by influencing the transcription of numerous genes involved in nutrient metabolism [75,76]. However, as we observed in the present study, the expression of the *PPARγ* gene was not affected by any of the treatments, but the *PGC-1α* gene showed a drastic change in response to LPL supplementation. This indicates that the *PGC-1α* gene in different tissues can be considered as a gene downstream of other genes [77], as we showed in our previous studies in the C2C12 cell line and gastrointestinal tissue [44,45,46]. Therefore, *PGC-1α* gene expression was not affected by the increase or decrease in the expression of the *PPARγ* gene. 

Potentially, *PGC-1α* leads to fatty acid oxidation in the hepatocytes. The *PGC-1α* gene regulates the production of the C-Ⅱ apolipoprotein, which is involved in the production and secretion of VLDL [77,78]. The main effect of LPL was a significantly decreased serum TG concentration. Therefore, it is possible that LPL supplementation could have caused a positive change in the transcription of *PGC-1α*, leading to increased production of apolipoproteins. The current study has shown that LPL supplementation regulated the production of pancreatic lipase, which ultimately caused a decrease in triglyceride-related pancreatitis and increased hydrolysis of triglycerides in the intestinal lumen, resulting in better absorption of lipid derivatives [79]. 

According to the results, LPL increased the expression of the lipoprotein lipase gene in the NE + LPL diet. In contrast, the LE + LPL diet decreased the *lipoprotein lipase* gene expression compared to the LE diet. The transcription of the *Lipoprotein lipase* gene significantly increased in all of the groups, as compared to the NE group. The NE + LPL and the LE groups had higher expression among other experimental groups (*p* < 0.01) (Figure 1B). The basal role of lipoprotein lipase is catalyzing the hydrolysis of the fatty acid component and lipoproteins, therefore providing non-esterified fatty acids for cell usage [80]. The expression of the *lipoprotein lipase* gene is high in capillaries and also in the liver of newly hatched chickens [81]. Additionally, nutritional and other physiological changes could contribute to the variation in lipoprotein lipase expression [82]. This enzyme needs cofactors such as phospholipids and Apo-protein C-Ⅱ to function [83]. The lipoprotein lipase enzyme causes the hydrolysis of triacylglycerol in the bloodstream, resulting in a reduction of triglycerides in the serum profile. This reduction is partially attributed to the activity of this enzyme [84]. In other words, by providing cofactors for this enzyme, LPL causes more consumption of VLDL and LDL by other tissues, including the liver itself. Therefore, the insignificant differences in *PPARγ* gene expression among the treatments, besides an increase in *PGC-1a* and lipoprotein lipase gene expression, result in increased TG usage in the liver and a lack of abdominal fat formation in the broilers.

LPL increased the *LPCAT3* transcription in the NE + LPL diet, while in the LE diet, the *LPCAT3* gene expression decreased with LPL supplementation. The expression of the *LPCAT3* gene increased in NE + LPL and LE birds, compared to the NE group (*p* < 0.01). Also, the transcription of the *LPCAT3* gene in the LE + LPL diet increased when compared to the NE group (*p* < 0.05) (Figure 1C). The LPC in the intestinal epithelial cells leads to an increment of lipid absorption. During this process, transcription of the *LPCAT3* gene is increased, resulting in the re-esterification of LPC to phosphatidylcholine [85]. *LPCAT3* also esterifies lysophospholipid species [86], converting 1-acyl LPC to phosphatidylcholine [87], 1-acyl lysophosphatidylserine to phosphatidylserine [88], and 1-acyl lysophosphatidylethanolamine to phosphatidylethanolamine. Moreover, the *LPCAT3* gene acts as a major LPC O-acyltransferase in the liver and intestine [89]. It also increases membrane dynamics and enables the transfer of triacylglycerols to nascent, very low-density lipoprotein (VLDL) particles [90]. However, previous studies demonstrated that a reduction of *LPCAT3* expression can decrease cholesterol, phospholipids, and plasma TG concentration [91]. Therefore, we expected that the utilization of LPL would increase the *LPCAT3* gene expression. Previous studies have shown that reducing the *LPCAT3* gene expression can increase the rate of apoptosis in liver cells [85]. The reason could be that by reducing the expression of *LPCAT3*, the amount of LPC esterification decreases. The amount of storage of fatty acids in liver cells probably increases since LPC is known to be effective in inducing cell death in liver cells [92]. In our study, we observed that the reduction of dietary energy and the inclusion of LPL in the diet increased the *LPCAT3* gene transcription, which might have reduced the fatty liver syndrome and apoptosis rate in liver cells. 

Based on our results, there was no significant difference in the expression of the RBP gene between dietary treatments. Although the amount of albumin protein remained unchanged across treatments, this may suggest the involvement of free fatty acids in the formation of chylomicrons in intestinal epithelial cells [93]. Moreover, the function of RBP protein depends on pre-albumin, as the RBP-pre-albumin complex transports the absorbed retinol and fatty acids from the liver to the various tissues through the bloodstream. Also, the dietary treatments did not induce any change in albumin and *RBP* gene expression, which suggests that there was no change in the amount of lipolysis in tissues such as abdominal fat tissue, although newly hatched chickens do not have abdominal fat tissue [94]. However, previous studies have shown that retinol, which regulates *RBP* gene expression, could potentially activate the transcription of the *TGF-β* gene. This interaction could prevent various diseases and improve immune system function [95,96]. The *CPT1A* gene expression response was affected by LPL inclusion in the diet. In the NE + LPL and LE + LPL birds, the transcription of the *CPT1A* markedly increased, as compared to the NE and LE groups (*p* < 0.05). However, the inclusion of LPL in both NE and LE diets caused an increase in *CPT1A* gene expression, while this increase was more visible in the LE + LPL (*p* < 0.001) (Figure 1D). The protein resulting from the transcription of the *CPT1A* gene is involved in the pathway of fatty acid beta-oxidation, which is part of lipid metabolism. Carnitine acetyl-transferase enzymes need a sufficient amount of L-carnitine to perform at their best [97]. L-carnitine is supplied through the diet, although it is also produced endogenously in the body from precursors such as lysine and methionine. The high expression of the *CPT1A* gene under the influence of treatments in all groups compared to the NE group can indicate the proper balance of these two amino acids in the diet. Also, studies have shown that *PGC-1α* gene transcription acts as an upstream regulator of the *CPT1A* gene and causes an increase in the *CPT1A* gene expression [98]. As observed in the present research, adding LPL to the breeders’ diet increased the *PGC-1α* and, ultimately, *CPT1A* gene expression in the offspring hepatocytes. While high-fat diets increase the *CPT1A* gene expression [99], starvation also increases the *CPT1A* gene expression. Moreover, lipoproteins can also increase the *CPT1A* gene transcription [100]. Thus, this gene is essential for the mitochondrial uptake of long-chain fatty acids and their subsequent β-oxidation in the mitochondria. Therefore, the *CPT1A* gene expression can be significantly increased under the influence of genetic factors such as increased expression of *PGC-1α* and *PPAR* families, as well as physiological factors such as apolipoproteins and nutritional factors such as dietary fat. 

### 3.5. Expression of Candidate Genes in the Jejunum

**Figure 2:**
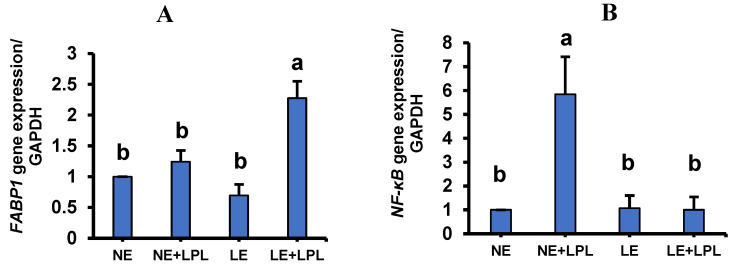
The interaction of energy and LPL supplement the expression of the genes in the intestine tissue. (**A**) Fatty Acid Binding Protein 1 (*FABP1*). (**B**) Nuclear Factor Kappa B (*NF-κB*). Whiskers represent SEM. ^ab^ Values within a column followed by different superscripts are significantly different. *p* < 0.05; Tukey’s pairwise comparison. NE: normal energy, LE: low energy, LPL: Lysophospholipid supplementation.

The liver fatty acid binding protein, also known as *FABP1*, is highly expressed in the duodenum and jejunum [101]. Compared to other FABPs, *FABP1* has a higher binding capacity for fatty acids and other lipid species, including LPLs [102]. It is also expressed in the intestinal epithelial and hepatocyte cells, where it plays a critical role in lipoprotein-mediated cholesterol uptake [103]. In addition, it binds to sterols [104], bilirubin [105], and free fatty acids [106] and, therefore, is involved in intracellular lipid transport. During fasting, the FABP1 is located on the apical surface of enterocytes [107]. This interaction results in a considerable up-regulation of the *FABP1* gene when LPL is supplemented. The addition of LPL in the diets with lower metabolizable energy resulted in the greatest expression of *FABP1,* as compared to the other groups (*p* < 0.05). In contrast, the birds fed with NE + LPL did not have a significant difference in the expression of *FABP1* relative to the NE and LE groups (Figure 2A). Thus, probably due to the elevated availability of fatty acid during LPL consumption in the lumen, the highest concentration of FABP1 is around the endoplasmic reticulum and Golgi network. Interestingly, the expression of *FABP1* is regulated by the *PPARs* [101]. *FABP1* has a positive regulatory effect on fatty acid beta-oxidation and positive regulation of hydrolase activity [108]. Also, the increase in *FABP1* expression could lead to overexpression of the liver and intestinal inflammatory genes [109]. It is worth noting that the overload of free fatty acids in the enterocyte after the feeding could be cytotoxic to the enterocyte cells. Thus, the FABP1 protein reduces the apoptotic effect of FFA in the intestinal cells by binding to the FFA [110,111]. The LPL supplementation increased this positive effect of FABP1 through the increment of *FABP1* gene transcription [112].

In addition, the transcription of the *TGF-β* gene did not have any difference between experimental treatments. However, the LE and LE + LPL groups demonstrated a numerical decrease compared to the other groups. Based on our observations, the consumption of NE + LPL diet in the breeders caused an increase in *NF-κB* gene expression, an inflammatory factor in the offspring intestinal epithelial cells. This is due to the decrease in *NF-κB* gene expression resulting from the reduction of dietary energy and the volume of free fatty acids in the intestinal epithelial cells. Actually, NF-κB cluster components regulate the transcriptional function of several promoters of pro-inflammatory cytokines [113], immune receptor proteins [114], transcription factors [115], and adhesion factors that contribute to intestinal inflammation [116]. The TGF-β potentially could activate the NF-κB factor. Through the mitochondrial biogenesis increment, the fatty acid β-oxidation changes in the adipocytes, leading to the conversion of the white adipocytes to the brown adipocytes [117]. 

According to the interaction results, the expression of the *NF-κB* gene was only increased by adding LPL to the NE diet (*p* < 0.01). In addition, the main effect of the LPL supplementation was a tendency to increase the transcription of the *NF-κB* gene. Autotaxin leads to the production of LPA and choline by affecting the LPC [118]. The produced LPA causes oxidative stress, alteration of *PPARγ* transcription, and mitochondrial dysfunction, probably through the reduction of *PGC-1α* expression. The LPAs and even LPCs in various tissues, as well as adipocytes, liver, and ovary tissue [119], bind to the G-protein receptors and cause the NF-κB transcriptor activation. Finally, the activated NF-κB induces the expression of pro-inflammatory cytokines, which further stimulate the migration of macrophages and monocytes to the digestive tract, increasing systemic inflammation [120].

### 3.6. Expression of FABP4 Gene in the Pancreas

**Figure 3:**
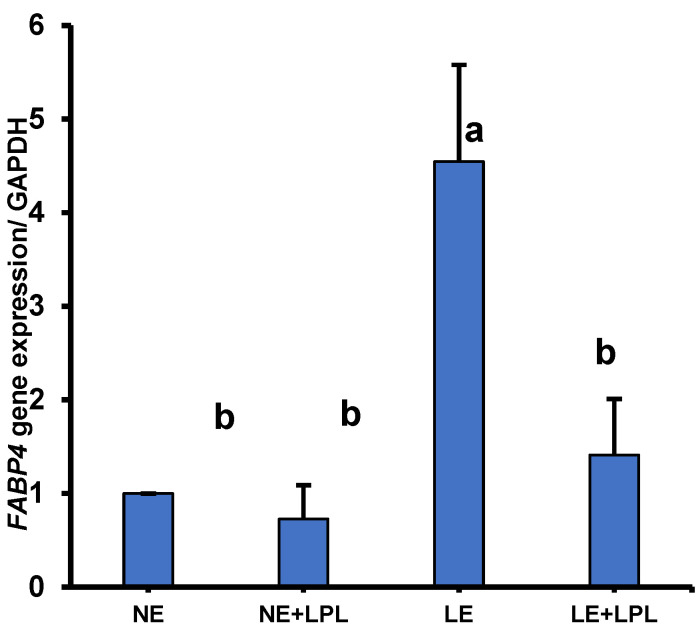
The interaction of energy and LPL supplement on the expression of the Fatty Acid Binding Protein 4 (*FABP4*) gene in the pancreas tissue. Whiskers represent SEM. ^ab^ Values within a column followed by different superscripts are significantly different. *p* < 0.05; Tukey’s pairwise comparison. NE: normal energy, LE: low energy, LPL: Lysophospholipid supplementation.

The interaction between metabolizable energy and LPL showed that *FABP4* gene expression in the offspring was higher in breeders fed with the LE diet (*p* < 0.01) (Figure 3). Moreover, the main effect of the energy showed that the LE diet had a tendency to increase the transcription of the *FABP4* gene (*p* = 0.08). Also, lysophospholipids could potentially bind to the albumin binding site in the same way as the long-chain fatty acids do [121]. With an increase in fatty acid absorption, the amount of lipoprotein secretion from the liver and the amount of β-oxidation of fatty acids in the mitochondria increases [122]. FABP4 is one of the key factors that demonstrate the abnormal positioning of fat sediment in non-adipose tissues, including the liver [123], and can also regulate enzyme activity [124]. In addition, the *FABP4* expression is regulated by the *PPARγ* [125]. It increases the hydrolytic activity of hormone-sensitive lipase [126], as well as regulating the transcription of *PPARγ* in the transport of specific *PPARγ* agonists to the nucleus. As lipolysis increases, the secretion of FABP4 protein from adipocytes also increases. This, in turn, enhances the activity of hormone-sensitive lipase, promoting the defective process and resulting in even more secretion of FABP4 protein [127].

## 4. Conclusions

Overall, our findings indicate that supplementation of LPL in broiler breeders’ diet with a low energy level favors offspring performance, serum metabolites, and hepatic endurance against oxidative stress. Therefore, the consumption of LPL by breeders improves the health and performance of the offspring, primarily through the regulation of genes involved in the offspring’s liver β-oxidation process. Overall, this study suggests that LPL is a safe and effective feed additive as it enhances lipid absorption and metabolism rate, leading to improved performance and health in poultry.

## Figures and Tables

**Table 1:** Ingredient composition and calculated nutrient content of the basal diets (as fed basis).

	Breeder Diets		Offspring Diet
Ingredients (g/kg)	Normal Diet	Low Energy Diet	Broiler Starter (1 to 7 d)
Corn	664.00	673.00	553.90
Soybean meal	177.00	176.00	384.00
Corn gluten meal	0.00	0.00	10.00
Wheat bran	38.00	38.00	0.00
Soybean oil	13.00	5.00	10.10
Limestone	82.00	82.00	11.30
Dicalcium phosphate	13.00	13.00	13.40
Sodium bicarbonate	2.50	2.50	2.20
NaCl	2.20	2.20	2.50
L-Lysine hydrochloride	-	-	2.20
DL-Methionine	1.60	1.60	3.40
L-Threonine	0.70	0.70	1.10
Choline chloride, 60%	1.00	1.00	0.80
Phytase ^1^	0.00	0.00	0.05
Multienzyme ^2^	0.00	0.00	0.10
Broiler vitamin-mineral premix ^3^	0.00	0.00	5.00
Breeder vitamin-mineral premix ^4^	5.00	5.00	0.00
Lysophospholipid	0.00	0.00	0.00
Total	1000	1000	1000
Calculated nutrients (%)			
Metabolizable Energy (kcal/kg)	2800	2760	3030
Crude protein	13.00	13.00	24.13
Digestible lysine	0.56	0.56	1.28
Digestible methionine + cysteine	0.54	0.54	0.95
Digestible threonine	0.47	0.47	0.86
Digestible isoleucine	0.43	0.43	0.87
Digestible valine	0.47	0.47	0.96
Ca	3.4	3.4	0.96
Available P	0.35	0.35	0.48
Na	0.18	0.18	0.16
Choline (mg/kg)	1200	1200	1455

^1^ Phytase: Ronozyme^®^ HiPhos 20,000 FYT/g (one FYT is defined as the amount of enzyme that releases 1 μmol of inorganic phosphate from phytate substrate per minute under reaction conditions with a phytate concentration of 5.0 mM/L at pH 5.5 and temperature 37 °C). ^2^ Multienzyme matrix value per kg: 500,000 kcal/kg metabolizable energy, 2000% protein, 80% lysine, 80% methionine + cysteine, and 80% threonine. ^3^ Vitamin and mineral supplied per kg diet: 12,000 IU Vitamin A, 5000 IU Vitamin D3, 80 IU Vitamin E, 3.2 mg vitamin K3, 3.2 mg vitamin B1, 65 mg niacin, 20 mg pantothenic acid, 3.4 mg vitamin B6, 0.22 mg biotin, 2.20 mg folic acid, 0.017 mg vitamin B12, 120 mg manganese, 110 mg zinc, 20 mg iron, 16 mg copper, 1.25 mg iodine, and 0.3 mg selenium. ^4^ Vitamin and mineral supplied per kg diet: 11,000 IU Vitamin A, 3500 IU Vitamin D3, 100 IU Vitamin E, 3.0 mg vitamin K3, 3.0 mg vitamin B1, 35 mg niacin, 15 mg pantothenic acid, 3.0 mg vitamin B6, 0.15 mg biotin, 1.50 mg folic acid, 0.02 mg vitamin B12, 120 mg manganese, 110 mg zinc, 40 mg iron, 16 mg copper, 1.25 mg iodine, and 0.3 mg selenium.

**Table 2:** Primers for Real Time-PCR assays.

Gene Name	Forward Primers (5′–3′)	Reverse Primers (5′–3′)	GenBank Accession No.	Product Length	Melting Temperature (Tm)	Reference
*PPARγ*	CATCAGGTTTGGGCGAATGC	TAACTGGTCGATGTCGCTGG	NM_001001460.2	76	60	[29]
*PGC-1α*	CATGTGCAACCAGGACTCTG	TGTCTGCATCCAGGTCGTTC	NM_001006457.2	131	59	[29]
*RBP*	TGGGAACGGGATGAAAGTGG	AGAGGAGGTGCTTGATTGCC	NM_205463.2	184	60	Present study
*CPT1A*	TGAGCACTCTTGGGCAGATG	TCTCCTTTGCAGTGTCCGTC	NM_001012898.1	108	60	Present study
*LPCAT3*	CCTCATCGTGTCCATCCTG	TGTACGACCCATAAGCCTCAG	XM_040661607.1	202	59	Present study
*TGF-β*	CTCGACACCGACTACTGCTT	TTCCACTGCAGATCCTTGCG	NM_001318456.1	95	60	Present study
*FABP1*	ACTGGCTCCAAAGAATGACCAATG	TGTCTCCGTTGAGTTCGGTCAC	NM_204192.4	162	61	Not published
*NF-κB*	TACTGATTGCTGCTGGAGTTGATGTC	TTGTGCCATCGTATGTAGTGCTGTC	NM_205134.2	156	63	[31]
*FABP4*	CTGGCCTGACAAAATGTGCG	CTTCCTGGTAGCAAACCCCA	NM_204290.2	109	60	[30]
*ASCL*	GCCAACAAGAAGATGAGCAAA	GGAGTTCATGTCGTGGGAGT	NM_204412.2	162	59	Present study
*LPL*	ACTTTTTCGCCGCTGCAC	CCCAGCTTTCATACATTCCTGTC	NM_205282.2	297	60	Present study
*GAPDH*	GAAGCTTACTGGAATGGCTTTCC	CGGCAGGTCAGGTCAACAA	NM_204305.2	66	60	[29]

**Table 3:** Growth performance of offspring (from 0 to 7 days of age) that hatched from breeders fed with dietary treatments for 8 and 12 weeks (57 and 61-week-old, respectively).

		57-Week-Old Breeders				61-Week-Old Breeders			
Main Effects		ABW ^1^ (g)	ADG ^2^ (g)	ADFI ^3^ (g)	FCR ^4^	ABW (g)	ADG (g)	ADFI (g)	FCR
Energy (kcal/kg)									
2800		195.64 ^a^	21.90	21.72	0.98	205.77	23.24	22.44	0.95
2760		190.73 ^b^	21.36	21.29	0.99	205.62	23.27	22.14	0.94
LPL (g/kg)									
0		186.16 ^b^	20.62 ^b^	21.35	1.02 ^b^	199.57 ^b^	22.57 ^b^	21.97	0.96 ^b^
0.5		200.21 ^a^	22.65 ^a^	21.66	0.95 ^a^	211.81 ^a^	24.13 ^a^	22.61	0.93 ^a^
**Interactions**									
Energy (kcal/kg)	LPL ^5^ (g/kg)								
2800	0	188.66	20.83	21.43	1.02	198.97	22.56	22.02	0.97
	0.5	202.62	22.97	22.01	0.95	212.57	24.29	22.87	0.93
2760	0	183.65	20.40	21.26	1.03	200.18	22.58	21.93	0.96
	0.5	197.81	22.32	21.32	0.95	211.05	23.97	22.35	0.92
SEM		1.77	0.24	0.25	0.009	1.07	0.18	0.27	0.92
***p*-Value**									
Energy		0.02	0.07	0.16	0.60	0.90	0.50	0.36	0.29
LPL		0.0001	0.0001	0.30	0.0001	0.0001	0.0001	0.06	0.002
Energy × LPL		0.96	0.72	0.40	0.60	0.29	0.46	0.51	0.75

^ab^ Values within a column followed by different superscripts are significantly different. *p* < 0.05; Tukey’s pairwise test. ^1^ Average body weight. ^2^ Average daily gain. ^3^ Average daily feed intake. ^4^ Feed conversion ratio. ^5^ Lysophospholipid.

**Table 4:** Blood biochemical parameters of day-old offspring of the second hatch, which hatched from breeders fed for 12 weeks (61-week-old) with dietary treatments.

Main Effects		TG (mg/dL) ^1^	Cholesterol (mg/dL)	ALT ^2^ (U/L)	AST ^3^ (U/L)	ALP ^4^ (U/L)	Albumin (g/dL)
Energy (kcal/kg)							
2800		91.14	549.82 ^a^	34.35	216.50 ^b^	3142.85	0.88
2760		96.28	504.62 ^b^	39.00	269.59 ^a^	3046.00	0.84
LPL (g/kg)							
0		106.28 ^a^	527.13	38.21	251.19 ^a^	3055.00	0.86
0.5		81.14 ^b^	527.36	35.14	235.00 ^b^	3133.85	0.86
**Interactions**							
Energy (kcal/kg)	LPL ^5^ (g/kg)						
2800	0	106.57	553.70	37.85	221.44	2782.85 ^c^	0.91
	0.5	75.71	546.04	30.85	211.70	3502.85 ^a^	0.85
2760	0	106.00	500.50	38.57	280.84	3327.14 ^b^	0.81
	0.5	86.57	508.72	39.42	258.23	2764.85 ^c^	0.87
SEM		4.54	15.65	2.97	4.57	247.76	0.04
***p*-Value**							
Energy		0.34	0.02	0.19	0.0001	0.74	0.46
LPL		0.0001	0.99	0.38	0.006	0.78	1.00
Energy × LPL		0.29	0.67	0.27	0.24	0.03	0.32

^abc^ Values within a column followed by different superscripts are significantly different. *p* < 0.05; Tukey’s pairwise test. ^1^ Triglyceride. ^2^ Alanine aminotransferase. ^3^ Aspartate aminotransferase. ^4^ Alkaline phosphatase. ^5^ Lysophospholipid.

**Table 5:** The hepatic antioxidative capability of day-old offspring of the second hatch, which hatched from breeders fed for 12 weeks (61-week-old) with dietary treatments.

Main Effects		SOD ^1^ (U/mg)	GPx ^2^ (U/mg)	TP ^3^ (U/mg)	TAC ^4^ (U/mg)	MDA ^5^ (mmol/mg)
Energy (kcal/kg)						
2800		240.39	66.92	15.35	2.48	146.50
2760		232.92	66.50	17.07	2.61	138.57
LPL (g/kg)						
0		259.00 ^a^	64.57 ^b^	15.50	2.58	142.14
0.5		214.32 ^b^	68.85 ^a^	16.92	2.51	142.92
**Interactions**						
Energy (kcal/kg)	LPL ^6^ (g/kg)					
2800	0	223.57 ^c^	63.42	14.57	2.43	134.28 ^c^
	0.5	257.21 ^b^	70.42	16.14	2.54	158.71 ^a^
2760	0	294.42 ^a^	65.71	16.42	2.73	150.00 ^b^
	0.5	171.42 ^d^	67.28	17.71	2.49	127.14 ^d^
SEM		11.96	1.57	1.00	0.18	8.48
***p*-Value**						
Energy		0.59	0.81	0.16	0.56	0.43
LPL		0.004	0.02	0.23	0.77	0.93
Energy × LPL		0.0001	0.15	0.90	0.42	0.02

^abcd^ Values within a column followed by different superscripts are significantly different. *p* < 0.05; Tukey’s pairwise test. ^1^ Superoxide dismutase. ^2^ Glutathione peroxidase. ^3^ Total protein. ^4^ Total antioxidative capacity. ^5^ Malondialdehyde. ^6^ Lysophospholipid.

## Data Availability

The data presented in this study can be accessed by contacting the corresponding author.
